# A novel murine model of autoimmune dysautonomia by α3 nicotinic acetylcholine receptor immunization

**DOI:** 10.3389/fnins.2022.1006923

**Published:** 2022-11-23

**Authors:** Makoto Yamakawa, Shunya Nakane, Eikichi Ihara, Nozomu Tawara, Hiroko Ikeda, Yoko Igarashi, Yoshihiro Komohara, Koutaro Takamatsu, Tokunori Ikeda, Yusuke Tomita, Shoichi Murai, Yukio Ando, Akihiro Mukaino, Yoshihiro Ogawa, Mitsuharu Ueda

**Affiliations:** ^1^Department of Neurology, Graduate School of Medical Sciences, Kumamoto University, Kumamoto, Japan; ^2^Department of Molecular Neurology and Therapeutics, Kumamoto University Hospital, Kumamoto, Japan; ^3^Department of Medicine and Bioregulatory Science, Graduate School of Medical Sciences, Kyushu University, Fukuoka, Japan; ^4^Department of Cell Pathology, Graduate School of Medical Sciences, Faculty of Life Sciences, Kumamoto University, Kumamoto, Japan; ^5^Department of Medical Information Sciences and Administration Planning (Biostatistics), Kumamoto University Hospital, Kumamoto, Japan; ^6^Laboratory of Clinical Pharmacology and Therapeutics, Faculty of Pharmaceutical Sciences, Sojo University, Kumamoto, Japan; ^7^Department of Respiratory Medicine, Graduate School of Medical Sciences, Kumamoto University, Kumamoto, Japan

**Keywords:** autoimmune dysautonomia, nicotinic AChR, murine model, immunization, autoantibody

## Abstract

We aimed to establish a novel murine model of autoimmune autonomic ganglionopathy (AAG), which represents autoimmune dysautonomia, associated with MHC class II to understand its pathomechanism and the pathogenicity of nicotinic acetylcholine receptor (nAChR) antibodies. The amino acid sequence of the mouse nAChRα3 protein was analyzed using an epitope prediction tool to predict the possible MHC class II binding mouse nAChRα3 peptides. We focused on two nAChRα3 peptides in the extracellular region, and experimental AAG (EAAG) was induced by immunization of C57BL/6 mice with these two different peptides. EAAG mice were examined both physiologically and histologically. Mice with EAAG generated nAChRα3 antibodies and exhibited autonomic dysfunction, including reduced heart rate, excessive fluctuations in systolic blood pressure, and intestinal transit slowing. Additionally, we observed skin lesions, such as alopecia and skin ulcers, in immunized mice. Neuronal cell density in the sympathetic cervical ganglia in immunized mice was significantly lower than that in control mice at the light microscopic level. We interpreted that active immunization of mice with nAChRα3 peptides causes autonomic dysfunction similar to human AAG induced by an antibody-mediated mechanism. We suggested a mechanism by which different HLA class II molecules might preferentially affect the nAChR-specific immune response, thus controlling diversification of the autoantibody response. Our novel murine model mimics AAG in humans and provides a useful tool to investigate its pathomechanism.

## Introduction

Autoimmune autonomic ganglionopathy (AAG) is an acquired autoimmune disease of the autonomic nervous system (Vernino et al., 2000, 2008). Human AAG (hAAG) is characterized by autonomic dysfunction, including sympathetic failure (e.g., orthostatic hypotension and anhidrosis) and parasympathetic failure (e.g., abnormal pupillary response, fixed heart rate, and dry mouth and eyes) (Winston and Vernino, 2009; Nakane et al., 2020). In a previous study, we reported that approximately 80% hAAG patients exhibit extra-autonomic manifestations, including the involvement of the central nervous system, sensory disturbance, endocrine disorders, autoimmune diseases, and tumors (Nakane et al., 2017). Autoantibodies (Abs) that target the ganglionic α3 nicotinic acetylcholine receptor (nAChR) are found in the sera of roughly half hAAG cases. Abs impair the autonomic ganglionic synaptic transmission (Wang Z. et al., 2010). We previously reported an association between ganglionic nAChR Abs and HLA-DRB1 alleles in the Japanese population (Maeda et al., 2016).

Currently, only one accepted animal model of active immunization with hAAG exists, which is the experimental autoimmune autonomic neuropathy (EAAN) model. This model is established by immunizing rabbits with a recombinant nAChRα3 subunit fusion protein derived from cDNA encoding residues 1–205 of the human nAChR α3 (Lennon et al., 2003; Vernino et al., 2003). Lennon et al. (2003) and Vernino et al. (2003) reported that EAAN rabbits presented prominent gastrointestinal dysmotility, urinary retention, impaired pupillary light reflex, reduced lacrimation, hypotension, impaired heart rate variability, and low plasma catecholamine levels. By investigating the effects of EAAN rabbit IgG passive transfer to mice, they concluded that Abs against the nAChRα3 subunit play a pathogenic role in EAAN by impairing autonomic synaptic transmission, and specifically considered cross-linking, internalization, and degradation of postsynaptic nAChRs as the pathological mechanism (Lennon et al., 2003; Vernino et al., 2003).

However, the region that serves as the antigen recognition site has not been identified in the rabbit EAAN model because immunization is performed using a recombinant protein corresponding to the N-terminal extracellular domain of the nAChRα3 subunit. Here, we established a novel murine model of hAAG by actively immunizing mice using two different nAChRα3 peptides. We conducted experiments using a reliable immune epitope prediction tool which could verify whether a recombinant protein containing the region that was predicted to serve as the antigen recognition site could be used to establish a murine model. The immunized mice exhibited autonomic dysfunction with poor body weight gain, which has been observed in the rabbit EAAN model and patients with AAG. Furthermore, we pathohistologically examined the autonomic ganglia to evaluate the pathological roles of the anti-nAChRα3 Abs in mice.

## Materials and methods

### Preparation of nAChRα3 peptides

**FIGURE 1:**
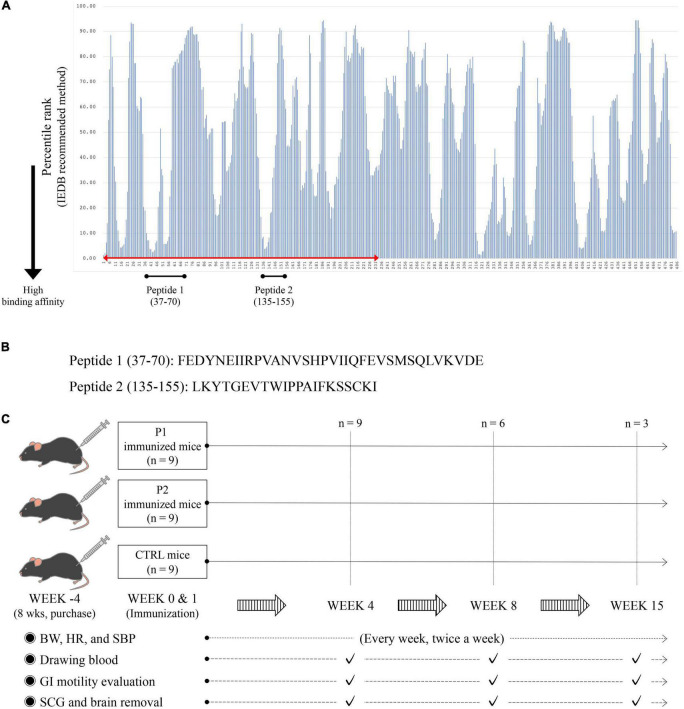
MHC H-2-I binding mouse nAChRα3 peptides predicted by immune epitope database (IEDB) analysis resource and research protocols. **(A)** The amino acid sequence of the mouse nAChRα3 protein was analyzed using IEBD based on methods recommended by IEDB. Numbers on the horizontal axis indicate amino acid positions at the N-terminus of nAChRα3-derived 15-mer peptides. Lower percentile rank numbers indicate stronger binding affinity to MHC H-2-I molecules. **(B)** The 34-mer long peptide with high percentile ranks for MHC H-2-I molecules was synthesized (Peptide 1, P1). The 21-mer long peptide with high percentile ranks for MHC H-2-I molecules was synthesized (Peptide 2, P2). **(C)** Twenty-seven mice (nine control mice with CFA only, nine nAChRα3 P1 immunized mice, and nine nAChRα3 P2 immunized mice) were used in this study.

To predict possible the MHC class II (H-2-I)-binding mouse nAChRα3 peptides, we analyzed the amino acid sequence of the mouse nAChRα3 protein using method recommended by the immune epitope database (IEDB)^1^ (Wang P. et al., 2010; Tomita et al., 2013, 2014; Fleri et al., 2017). The program was used to analyze 15-amino acid long sequences offset to encompass the entire protein. We defined percentile scores of less than 10 as an indicator of stronger binding affinity for MHC H-2-I molecules and selected the regions predicted to have a high binding affinity to at least two frequently observed MHC H-2-I molecules (Figure 1A). The 34-mer peptide (peptide 1, P1), mouse nAChRα3 37–70 (FEDYNEIIRPVANVSHPVIIQFEVSMSQLVKVDE), was predicted to exhibit strong binding affinity to MHC H-2-I molecules encoded by the H-2-IAb genes. The 21-mer peptide (peptide 2, P2), mouse nAChRα3 135–155 (LKYTGEVTWIPPAIFKSSCKI), was predicted to exhibit a strong binding affinity for MHC H-2-I molecules encoded by H-2-IAb genes. Moreover, both peptides were identified as long peptides (Figures 1A,B). Given the predicted epitope affinities for MHC H-2-I molecules, in the current study, we focused on these two nAChRα3 long peptides in the extracellular region.

The two mouse nAChRα3 long peptides presented by MHC H-2-I were biochemically synthesized (GenScript, Piscataway, NJ, USA; HPLC purity = 98.1%).

### Mouse immunization using nAChRα3 peptides

All animal procedures were performed under specific pathogen-free conditions in accordance with the institutional guidelines and were approved by the Animal Care and Use Committee of Laboratory Animal Utilization of Kumamoto University (approval number: A2019-152). Female C57BL/6 mice (8 weeks old), purchased from Charles River Laboratories Japan Inc. (Yokohama, Japan), were used and had free access to food and water. At 12 weeks of age, we immunized the mice intradermally at the base of the tail using 200 μg nAChRα3 peptide (P1 and P2) emulsified in Freund’s complete adjuvant (CFA) containing 2 mg/mL heat-killed *Mycobacterium tuberculosis* H37RA (Becton, Dickinson and Company, Franklin Lakes, NJ, USA). Additionally, 12-week-old mice were administered 350 ng *Bordetella pertussis* toxin (List Biological Labs, Campbell, CA, USA) in 0.5 ml PBS intraperitoneally at week 0 (day 0) (Ikeda et al., 2010). All mice received equal volumes of recombinant nAChRα3 subunit emulsified in CFA at day 7 after immunization at 13 weeks of age (Figure 1C).

Twenty-seven mice (nine control mice receiving CFA only, nine nAChRα3 P1-immunized mice, and nine nAChRα3 P2-immunized mice) were used in the present study (Figure 1C).

### Dot blot assay to detect nAChRα3 antibodies

After terminal anesthesia, blood was collected by cardiac puncture into tubes containing heparin at week 4 (day 30 after immunization), 8 (day 60 after immunization), and 15 (day 100 after immunization) (Figure 1C). Blood samples were centrifuged at 4°C, and plasma samples were stored at −80°C.

The presence of nAChR-binding antibodies was determined using dot blot analysis. Nitrocellulose membranes (Bio-Rad, Hercules, CA, USA) were spotted with a 1.0 μg/μL RIPA) buffer solution of P1 or P2 peptide (1 μL/spot). After drying, the membranes were blocked with 5% non-fat dry milk in PBS for 1 h at room temperature and then washed thrice in PBS with 0.1% Tween (PBS-T). The membranes were incubated with a 1:100 dilution of mouse serum in 5% non-fat dry milk/PBS overnight at 4°C. After washing thrice with PBS-T, the membranes were incubated with a horseradish peroxidase (HRP)-conjugated anti-mouse IgG antibody (Agilent Technologies, Santa Clara, CA, USA) at a 1:2000 dilution for 1 h at 4°C. Thereafter, the membranes were washed three times and incubated with ECL™ Prime Western Blotting System (GE Healthcare Life Sciences, Chicago, IL, USA) for 5 min. The light intensity of spots on the membrane was detected using a ChemiDoc Touch Imaging System (Bio-Rad).

### Body weight, systolic blood pressure, and heart rate measurements

The mice were gently restrained for all procedures and were acclimatized to handling in the research environment for four weeks prior to immunization. All measurements were performed while the mice were awake. Systolic blood pressure (SBP) and heart rate (HR) were measured using a tail-cuff plethysmograph using a blood pressure measuring system (MODEL MK-1030^®^; Muromachi Kikai, Tokyo, Japan), following the manufacturer’s protocol (Ueda et al., 2003; Suliburska et al., 2014). Blood pressure and pulse rate were measured at 37°C in an animal holder fabricated of dark brown acryl, allowing blood pressure measurement under relatively stress-free conditions. Continuous measurement was performed up to five times for each mouse. For these parameters, the average and standard deviation were calculated weekly for each group. The weights, SBP, and HR were measured twice a week, every week (Figure 1C).

All mice were examined for general health and neurological signs daily, as previously described (Nakane et al., 2003a,b). Mice from each group were evaluated at weeks 4, 8, and 15. We performed antibody testing, gastrointestinal motility studies, and histopathological evaluations of the brain and sympathetic cervical ganglia (SCG).

### Evaluation of gastrointestinal motility *in vivo*

Gastrointestinal (GI) motility was evaluated at weeks 4, 8, and 15 after pretreatment with P1, P2, or control (Figure 1C). The *in vivo* GI motility was assessed by the intestinal distribution of non-absorbable fluorescein-labeled dextran (70 kDa, FD70; Sigma-Aldrich, St Louis, MO, USA), as previously described with minor modifications (de Backer et al., 2008; Akiho et al., 2014).

FD70 (200 μL, 25 mg/mL) was orally administered 30 min before the mice were sacrificed by cervical dislocation. The abdomen was cut open, and the entire GI tract was excised from the lower part of the esophagus to the rectum. Immediately after the mesenteric membrane was removed, FD70 was visualized using ChemiDoc Touch (Bio-Rad). We captured two images of the GI tract—one using the normal illumination mode (Ponceau S filter; 590/110 nm, white epi mode) and one the fluorescent mode (Gel Green filter; 590/110 nm, *trans*-UV mode)—with an exposure time of 0.001 s. The merged image was then used for further analysis. The fluorescent intensity for the entire GI tract was determined using ImageJ 1.52a (National Institutes of Health, Bethesda, MD, USA). Data were expressed as the percentage of fluorescence intensity per segment of 14 parts (stom, stomach; sb, small bowel segments 1–10; cecum, col, colon segments 1–2) and plotted in a histogram. The histogram was quantified by the geometric center (GC), which was calculated using the following formula: S (% FD70 per segment*segment number)/100. The smaller the GC is, the more impaired gastrointestinal motility due to numbering 14 parts with the first part (stom) 1 and the final part (col2) 14. The GC has been used frequently and reliably to estimate GI transit. The geometric center (GC) was calculated using the following formula:


S(%FD70persegment×segmentnumber)/100.

GC is frequently and reliably employed to estimate GI transit (Dhaese et al., 2009; Akiho et al., 2014; Van Dingenen et al., 2018).

### Histopathological studies of the sympathetic cervical ganglia and brains

At weeks 4, 8, and 15, the mice were anaesthetized with sevoflurane inhalation and perfused with 4% paraformaldehyde via intracardiac puncture. The SCG and brains were dissected and assessed (Figure 1C and Supplementary Data 1; Ikegami et al., 2004; Yang et al., 2010).

Dissociated SCG were fixed in 4% paraformaldehyde for 24 h at 4°C, dehydrated, embedded in paraffin, and sectioned at a thickness of 4 μm. SCG sections were stained with hematoxylin and eosin or were labeled using Abs specific for the nAChRα3 subunit. In this analysis, we confirmed that staining with antibodies specific for synaptophysin (R&D Systems, Minneapolis, MN, USA), a neuronal marker, is consistent with staining with antibodies specific for nAChRα3. Antigen retrieval for sections stained for nAChRα3 subunits was performed by incubation of sections in citrate buffer solution (pH 6) for 30 min at 95°C. The sections were incubated for 30 min in blocking buffer (Agilent Technologies, Santa Clara, CA, USA) and then placed in anti-nAChRα3 subunit antibody (Proteintech Group, Japan, Tokyo, Japan) solutions in PBS overnight at 4°C. Anti-nAChRα3 subunit antibodies were used at a dilution of 1:50. Following washes, the sections were incubated with anti-Rabbit IgG Alexa Fluor 488-conjugated antibody (Thermo Fisher Scientific, Waltham, MA, USA) for 1 h at 37°C. This secondary antibody was used at a 1:500 dilution. The sections were then washed and embedded under coverslips in Vector shield mounting medium with DAPI (Vector Laboratories Inc., Burlingame, CA, USA). Fluorescence images were captured using a fluorescence microscope and confocal microscope. The areas of SCG were measured using NIH Image software. The cells were selected for measurement if the nucleus was centrally located in the cytoplasm. We quantified changes in the number of immunostained cells by counting the number of nAChRα3 subunit-positive cells in the SCG. Data were averaged for groups of experiments, and the neuronal dell density (NCD) in SCG was expressed as No./mm^2^.

### Anti-P1 and P2 antibodies examined by enzyme-linked immunosorbent assay

After completing the animal model experiments, we established an ELISA for anti-P1 and -P2 antibodies in the serum from seropositive patients with hAAG. All participants provided written informed consent to participate in this assay. Ethical approval was granted by Kumamoto University Hospital (approval number 2056). In this study, 213 hAAG patients from throughout Japan were identified by a luciferase immunoprecipitation assay as having anti-gAChR Abs (Supplementary Data 2). In the present study, we investigated 109 subjects (mean age, 59.5 ± 20.6 years; 64 males and 45 females) who were seropositive for the anti-gAChRα3 Abs and who had sufficient serum volume left. As a control group, we included 32 healthy individuals (mean age, 32.7 ± 9.2 years; 21 males and 11 females).

Enzyme-linked immunosorbent assay was performed according to the following protocol: Nunc™ Microwell™ 96-well microplates (Nunc, Rochester, NY, USA) were coated with 50 ng P1 or P2, diluted appropriately in carbonate coating buffer (NaHCO_3_ 840 mg, Na_2_CO_3_ 360 mg, DW 100 ml), and were incubated at 4°C overnight. The plates were blocked with ChonBlock™ blocking/sample dilution buffer (Iwai Chemicals Co. Ltd., Tokyo, Japan) at room temperature for 1 h. After one wash with PBS-T (0.05%), serum from controls and AAG patients, diluted at 1:100 in ChonBlock™ Blocking/Sample Dilution Buffer, were added to the wells and the plates were incubated at 37°C for 4 h. Optimal serum binding was initially determined using a dilution curve of patient sera, ranging from 1:100, 1:500, 1:1000, and 1:10,000 dilutions. Following three washes with PBS-T (0.05%), 100 μl/well of horseradish peroxidase-conjugated rabbit anti-mouse IgG (1:1000 dilution) (Agilent Technologies, Santa Clara, CA, USA) was added to the plates, which were then incubated for 1 h at 37°C. After three washes with PBS-T (0.05%), the complexes formed in each well were incubated with 100 μl of KPL Sure Blue™ TMB Microwell Peroxidase Substrate (SeraCare Life Sciences, Milford, MA, USA) (#5120-0077). After 5 min, the reaction was stopped with 1 N HCl. The absorbance values (AVs) were measured at 450 nm using a Bio-Rad xMark™ Microplate Absorbance Spectrophotometer (Hercules, CA, USA). Based on the data from the 32 healthy controls, cut-off values were calculated as mean ± 2 standard deviations from the mean.

### Statistical analysis

Commercially available statistical software SigmaPlot^®^ (SPSS, Inc., Chicago, IL, USA) was used for data analysis. When comparing findings between the three groups (P1-immunized mouse group, P2-immunized mouse group, and control group), normally distributed data were analyzed via one-way analysis of variance (ANOVA). To compare non-normally distributed data between the three groups, we performed one-way ANOVA on ranks. For all analyses, the level of statistical significance was set at *p* < 0.05.

## Results

We established a nAChRα3-induced murine model to evaluate the autonomic dysfunction after confirming autoantibody production. We injected female B6 mice with 200 μg nAChRα3 peptide at weeks 0 and 1 (12 and 13 weeks of age). However, two mice from the control group died because of the CFA injection procedure. Based on the initial experimental protocol (Figure 1C), we then assessed nine P1-immunized mice, nine P2-immunized mice, and seven control mice.

### Dot blot assay for the detection of nAChRα3 antibodies

The results of the dot blot assay showed that Abs against nAChRα3 P1 or P2 were present in the sera of almost all immunized mice (16/18, 88.9%) (Supplementary Data 3). Anti-nAChRα3 P1 Abs were detected in all nine serum samples from the P1-immunized mouse group at each time point. In the P2-immunized mouse group, 100% mice (3 of 3) in week 4, 66.7% (2 of 3) in week 8, and 66.7% (2 of 3) in week 15 were positive for anti-nAChRα3 P2 Abs. In contrast, sera from the control mice exhibited no detectable anti-nAChRα3 P1 or P2 Abs (Supplementary Data 3).

### nAChRα3 immunization causes autonomic dysfunction with poor body weight gain

Nine mice from the P1-immunized group, nine from the P2-immunized group, and seven from the control group were evaluated at week 4. Six P1-immunized mice, six P2-immunized mice, and four control mice were evaluated at week 8. Three P1-immunized mice, three P2-immunized mice, and two control mice were evaluated at week 15.

**FIGURE 2:**
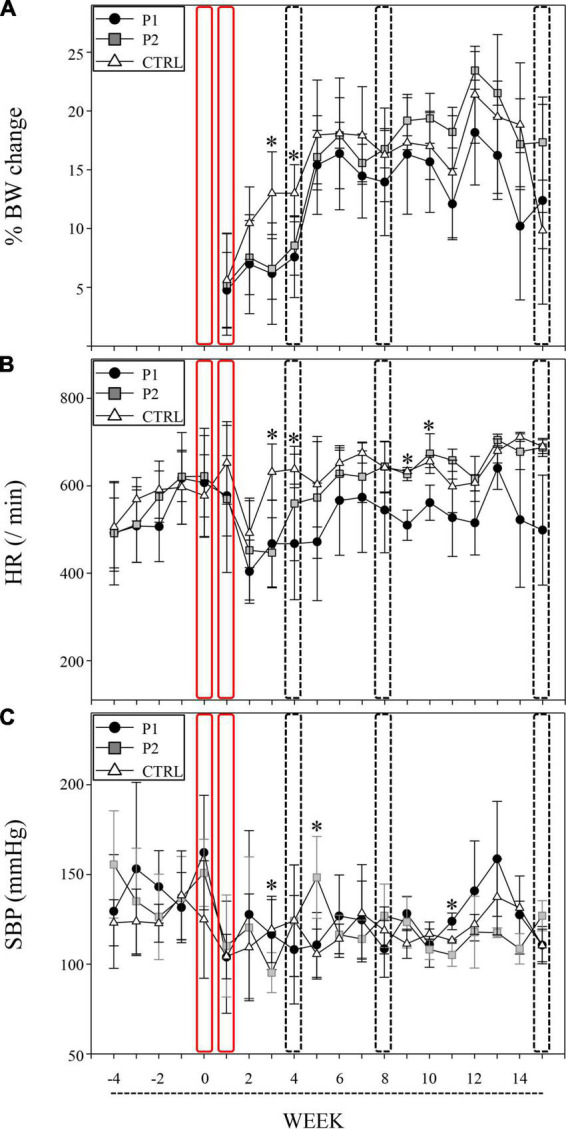
nAChRα3 peptides induced autonomic dysfunction with poor BW gain. Immunizations of nAChRα3 were performed at week 0 and 1 (red frame), and sacrifices were performed at week 4, 8, and 15 (frame of dotted line). Data are expressed as mean ± standard deviation. Asterisks denote statistical significance level (Supplementary Data 2). **(A)** Serial weight changes, **(B)** heart rate **(**HR, /min**)**, and **(C)** systolic blood pressure (SBP, mmHg) in immunized and control (CTRL) mice. Filled circles represent P1-immunized mice, gray squares represent P2-immunized mice, and open triangles represent CTRL mice.

The immunized mice significantly exhibited lower BW gain within four weeks of the first immunization compared to the controls. Similar BW gain was observed at weeks 3 and 4 in both the P1- and P2- immunized mice (*p* = 0.001 and *p* = 0.003, respectively; Figure 2A and Supplementary Data 4).

To assess the capacity for autonomic function in the cardiovascular system, we measured HR and SBP twice, every week. nAChRα3-immunized mice, including both P1 and P2 groups, exhibited significant reductions in HR, whereas the control mice did not exhibit any reduction in HR at week 3 (*p* < 0.001, Figures 2B,C and Supplementary Data 4). A significant reduction in HR in P1-immunized mice compared to P2-immunized mice and control mice was observed at week 4 (*p* = 0.021, Figure 2B and Supplementary Data 4). Furthermore, P2-immunized mice with poor weight gain exhibited characteristic SBP instability at weeks 3 and 5. The SBP of P2-immunized mice at week 3 was significantly lower than that of P1-immunized mice and control mice (*p* = 0.013, Figure 2C and Supplementary Data 4). At week 5, a higher SBP was observed in P2-immunized mice than in P1-immunized mice and control mice (*p* = 0.005, Figure 2C and Supplementary Data 4). P1-immunized mice continued to exhibit lower HR than P2-immunized mice and control mice at weeks 9 and 10 (*p* = 0.003 and 0.036, respectively; Figure 2B and Supplementary Data 4). P2-immunized mice still had lower SBP than P1-immunized mice and control mice at week 11 (*p* = 0.016, Figure 2C and Supplementary Data 4).

### Skin lesions in nAChRα3-immunized mice

**FIGURE 3:**
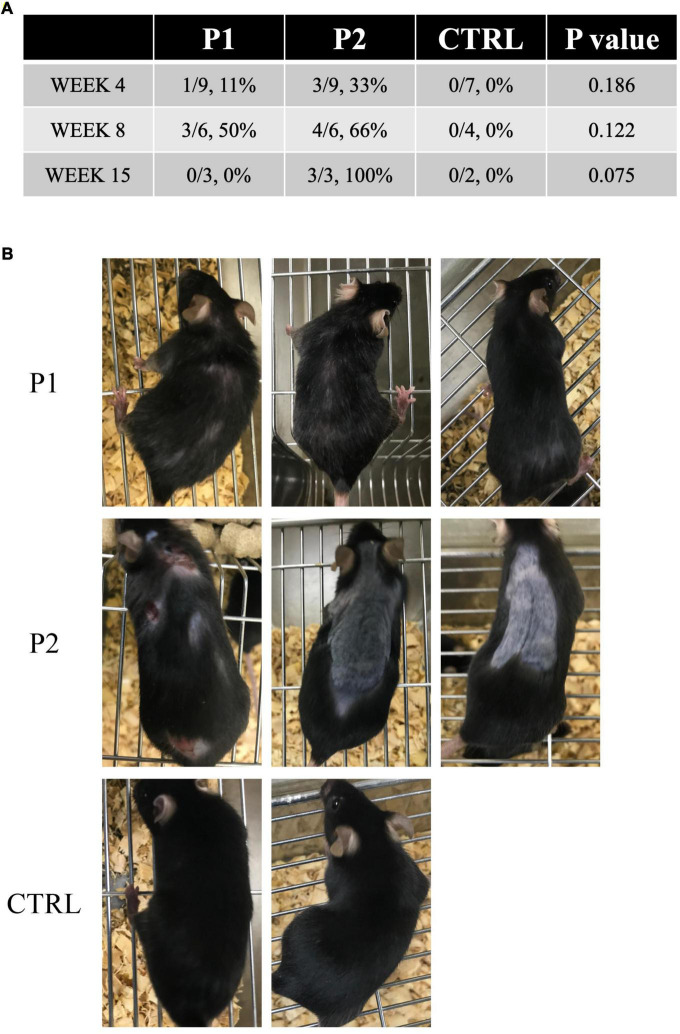
Skin lesions in immunized mice. **(A)** Prevalence of skin lesions in each group at each time point. **(B)** All mice at week 15. In P2 immunized mice, skin ulcers and alopecia were observed on the back of one mouse, whereas the other two mice exhibited considerable alopecia of the back.

We followed the chronological changes in the general health and neurological signs of all mice after immunization and observed no clinical neurological defects. However, the immunized mice demonstrated skin lesions, including skin ulcers and alopecia of the back (Figures 3A,B). Skin lesions were isolated to the injection site at the base of the tail in each immunized mouse. In contrast, all control mice remained healthy and did not exhibit any skin lesions, despite receiving CFA injections (Figure 3A). The first skin lesions were observed at week 4 in both groups of immunized mice and were more frequently observed in P1- and P2-immunized mice than in the control mice at week 8 (*p* = 0.122, Figure 3A). At week 15, the symptoms reached the maximum levels in P2-immunized mice (*p* = 0.075, Figure 3A). We observed three skin ulcers in the posterior region of the neck and back and two areas of alopecia on the back of one P2-immunized mouse. In addition, we recognized considerable alopecia on the back of two P2-immunized mice (Figure 3B).

### nAChRα3 immunization causes intestinal transit slowing

**FIGURE 4:**
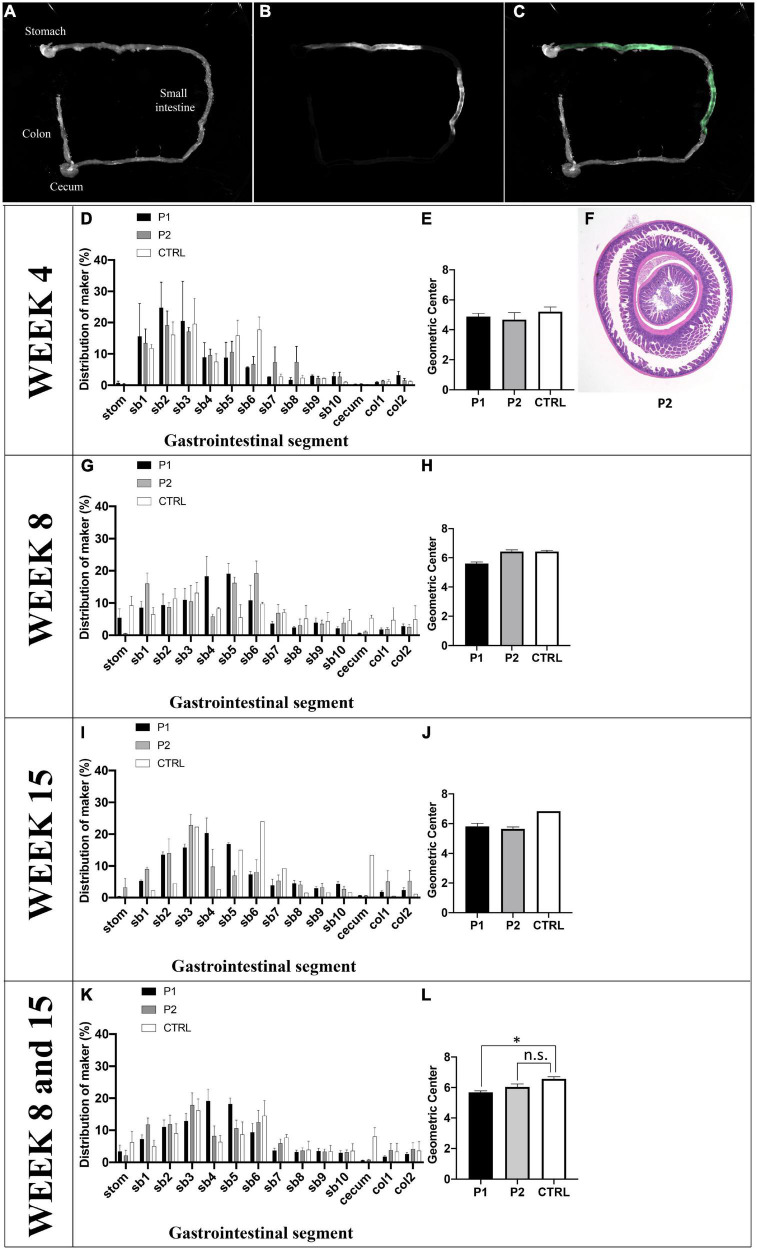
Quantification of intestinal transit. **(A)** Normal illumination mode, **(B)** fluorescent mode, and **(C)** merge of intestinal transit from one control mouse as a representative image. **(D–J)** GI motility *in vivo* was assessed by the intestinal distribution of non-absorbable fluorescein-labeled dextran (FD70). **(D)** At week 4, **(G)** week 8, and **(I)** week 15, the distribution patterns of FD70 was plotted in a histogram, indicating results from each group. **(E)** At week 4, **(H)** week 8, and **(J)** week 15, the geometric center (GC) of the mice are depicted using a histogram. **(F)** The histological findings of ileoileal intussusception in one mouse treated with peptide 1 on day 30 are shown. **(K)** The distribution patterns of FD70 of the three groups are plotted in a histogram, and **(L)** the GC values of the three groups are shown in a histogram. The fluorescent intensity for the entire GI tract was calculated using ImageJ 1.52a. Data are expressed as the percentage of fluorescence intensity per segment of 14 parts (stom, stomach; sb, small bowel segments 1–10; cecum, col, colon segments 1–2) and is plotted in a histogram. The GC was calculated using the following formula: S (% FD70 per segment × segment number)/100. Asterisk denotes statistical significance.

The effects of P1 or P2 immunization on GI motility were assessed *in vivo*. Seven P1-immunized mice (week 4, *n* = 2; week 8, *n* = 3; week 15, *n* = 2), eight P2-immunized mice (week 4, *n* = 2; week 8, *n* = 3; week 15, *n* = 3), and six control mice (week 4, *n* = 3; week 8, *n* = 2; week 15, *n* = 1) were evaluated (Figures 4D,G,I,K). We observed bimodal peaks in the GI fluorescence distribution in the control mice, and GI motility tended to increase weekly with increasing age of the control mice (Figures 4A–C).

The mean geometric center (GC) values at weeks 4, 8, and 15 were 5.21 ± 0.30 (*n* = 3), 6.44 ± 0.08 (*n* = 2), and 6.84 (*n* = 1), respectively (Figures 4E,H,J). At week 4, there was no difference in GC among the three groups; however, one mouse immunized with P2 exhibited ileoileal intussusception (Figure 4F). At week 8, GI motility was altered in P1-immunized mice. The mean value of GC in P1-immunized mice (5.61 ± 0.11, *n* = 3) was lower than that observed in control mice (6.44 ± 0.08, *n* = 2). GI motility was altered in P2-immunized mice at week 15. The mean value of GC in P2-immunized mice (5.65 ± 0.14, *n* = 3) was lower than that in control mice (6.84, *n* = 1). When we compared mice between weeks 8 and 15, GI motility was altered in P1-immunized mice, and it also appeared to be altered in P2-immunized mice (Figure 4H). The mean values of GC in P1- (5.69 ± 0.10, *n* = 5) and P2-immunized mice (6.04 ± 0.19, *n* = 6) were significantly different (*p* = 0.022) and tended to be (*p* = 0.110) lower than those of control mice (6.57 ± 0.14, *n* = 3) (Figure 4L).

### Histopathological studies of the sympathetic cervical ganglia and brains

We determined the contribution of nAChRα3 immunization in the regulation of autonomic dysfunction following two challenges with nAChRα3 peptides. We analyzed the SCG and brain samples obtained from the immunized and control mice for NCD in SCG and the presence or absence of inflammation in the SCG and brain at weeks 4, 8, and 15. We were unable to evaluate the SCG neuronal density of all mice because it was technically difficult to dissociate SCG from the neck of each mouse.

**FIGURE 5:**
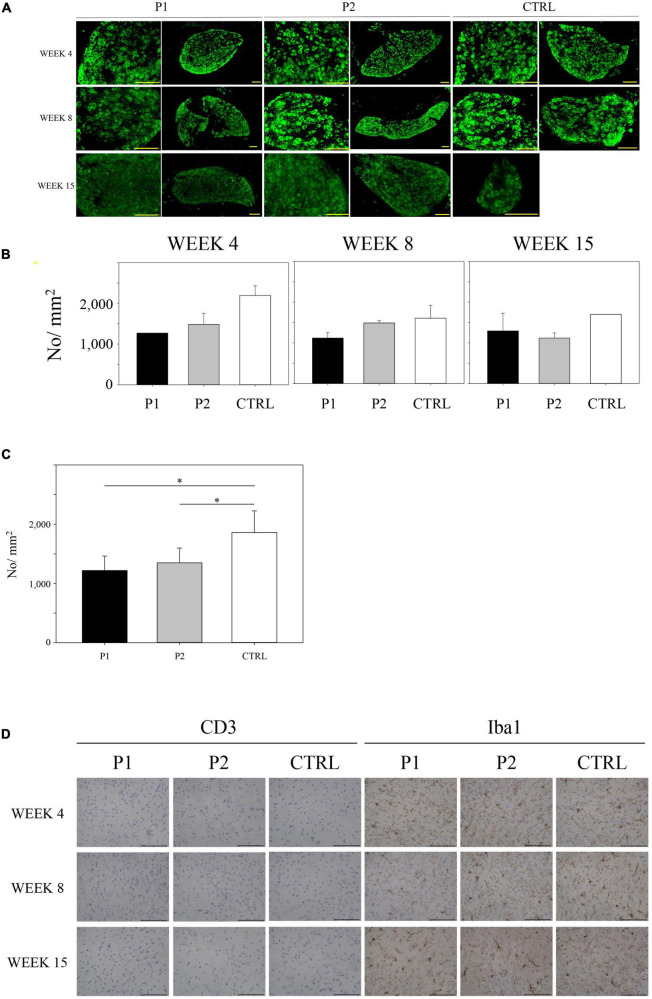
Immunohistochemical studies on sympathetic cervical ganglia (SCG) and brain. **(A)** Single-labeled using antibodies specific for the nAChRα3 subunit of each group at each timepoint. **(B)** Neuronal cell density (NCD) in SCG at weeks 4, 8, and 15. **(C)** The mean No./mm2 value for the control mice across all time points was 1860.3 ± 364.5 (*n* = 5). This level was significantly higher than that obtained for other groups because the No./mm2 values totaled 1217.8 ± 246.2 (*n* = 5) and 1348.4 ± 249.6 (*n* = 8) for P1- and P2-immunized mice, respectively (*p* = 0.006 by one-way ANOVA). All bar graphs represent means ± standard deviation. **(D)** Immunohistochemical studies of brain CD3 and Iba-1. Scale bar: 200 μm. Asterisk denotes statistical significance.

Sympathetic cervical ganglia from immunized mice appeared normal, and no inflammation was observed. We noticed that both groups of immunized mice exhibited a reduction in NCD at weeks 4, 8, and 15 (Figures 5A,B). Mean No./mm^2^ values in P1-immunized mice, P2-immunized mice, and control mice at week 4 were 1268.7 (*n* = 1), 1484.0 ± 271.1 (*n* = 3), and 2193.3 ± 237.0 (*n* = 2), respectively, those at week 8 were 1118.1 ± 135.5 (*n* = 2), 1489.6 ± 63.5 (*n* = 2), and 1606.5 ± 315.6 (*n* = 2), respectively, and those at week 15 were 1292.1 ± 436.5 (*n* = 2), 1118.7 ± 124.4 (*n* = 3), and 1701.7 (*n* = 1), respectively. The mean No./mm^2^ value for the control mice across all weeks was 1860.3 ± 364.5 (*n* = 5). This level was significantly higher than that obtained for other groups because the No./mm^2^ values totaled 1217.8 ± 246.2 (*n* = 5) and 1348.4 ± 249.6 (*n* = 8) for P1- and P2-immunized mice, respectively (*p* = 0.006, Figure 5C).

We next examined the brains of P1-immunized, P2-immunized, and control mice for signs of pathology. IHC staining for CD3 and Iba-1 was performed to evaluate inflammation in the brain. We observed low levels of CD3^+^ lymphocyte infiltration in the brain tissue. Although microglia were detected in the brain, microglial density did not differ between the P1-immunized, P2-immunized, and control mice at weeks 4, 8, and 15 (Figure 5D).

### Anti-P1 and P2 antibodies examined by enzyme-linked immunosorbent assay

**FIGURE 6:**
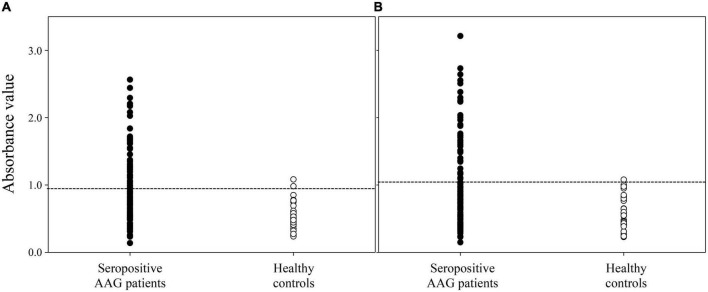
Detection of anti-P1 and P2 antibodies by ELISA. We tested sera from seropositive patients with human autoimmune autonomic ganglionopathy (hAAG) and healthy controls. The results of a test based on the ELISA show that 45.0% (49 of 109) of the sera from seropositive patients with hAAG are positive for anti-P1 and/or P2 Abs. **(A)** The anti-P1 Abs were detected in 43 samples (39.4%) from patients with hAAG, conversely, these Abs were detected in only two sample (6.3%) from healthy controls (*p* < 0.001). The mean anti-P1 level in healthy controls is 0.522 absorbance value (AV), which is significantly different from the value for the samples from patients with hAAG, which have a mean titer of 0.927 AV (*p* < 0.001). **(B)** The anti-P2 Abs were detected in 40 samples (36.7%) from patients with hAAG conversely, these Abs were detected in only one sample (3.1%) from healthy controls (*p* < 0.001). The mean anti-P2 level in healthy controls is 0.573 AV, which is significantly lower than the value for the samples from patients with hAAG, which have a mean level of 1.029 AV (*p* < 0.001).

The ELISA assay showed that anti-gAChRα3 and anti-P1 and/or -P2 Abs were detected in the serum of three healthy volunteers (3/32, 9.4%). In contrast, 45.0% (49/109) of sera from seropositive patients with hAAG were positive for anti-P1 and/or -P2 Abs (*p* < 0.001) (Figure 6). The mean levels of anti-P1 Abs in seropositive patients with hAAG and in healthy controls were 0.927 AV and 0.522 AV (*P* < 0.001), and the mean levels of anti-P2 Abs in seropositive patients with hAAG and in healthy controls were 1.029 AV and 0.573 AV (*P* < 0.001), respectively. More specifically, only anti-P1 Abs were detected in 10 samples (9/49, 18.4%), and only anti-P2 Abs were detected in seven samples from the hAAG group (6/49, 12.2%). Furthermore, 34 of the 49 samples (69.4%) from patients with hAAG were positive for both Abs.

## Discussion

In hAAG, pathogenic Abs target nAChRs in the autonomic ganglia. Serum from patients with hAAG contains anti-ganglionic nAChR Abs, which have been shown to interfere with synaptic transmission *in vitro*, as reported in previous analyses (Wang et al., 2007; Vernino et al., 2008; Wang Z. et al., 2010). In the current study, we demonstrated that the anti-nAChRα3 Abs generated via active immunization with the extracellular region of mouse nAChRα3 caused autonomic dysfunction. The murine model we developed is the first one based on active immunization; however, as already mentioned, there is a previous murine model based on passive transfer (Vernino et al., 2004).

In our study, we referred to the theory of experimental autoimmune encephalomyelitis (EAE) and experimental autoimmune myasthenia gravis (EAMG) induction through immunization (Stromnes and Goverman, 2006; Baxter, 2007; Baggi et al., 2012; Mantegazza et al., 2016). The hAAG animal model was first described in 2003 (Lennon et al., 2003; Vernino et al., 2003), when a clinical form of AAG was induced in rabbits via active immunization with nAChRα3 fusion proteins. We conducted experiments with the goal of establishing a new murine model of hAAG via active immunization. In the rabbit model by active immunization described above, a relatively large protein, the 1–205 region of nAChRα3, was used as an antigen (Lennon et al., 2003; Vernino et al., 2003). In the current study, we used the IEDB to identify regions within the extracellular domain of nAChR that are likely to act as MHC class II antigen recognition sites. There were three main reasons why we set MHC class II antigen recognition in the epitope prediction tool in IEDB: (1) MHC class II is necessary to trigger antibody production through the activation of B cells (Yuseff et al., 2013); (2) we previously presented evidence that the autoimmune response to ganglionic nAChR is influenced by HLA-DRB1*04:03 alleles (Maeda et al., 2016); and (3) several studies have indicated that a relationship exists between HLA-DRB1 alleles and autoantibodies, such as shared epitope-encoding DRB1 alleles and anti-citrullinated peptide antibodies in rheumatoid arthritis (Gonzalez-Gay et al., 2002; Reveille, 2006; Holoshitz, 2010). Based on the analysis results from the epitope prediction tool, we selected two regions, and immunized the mice with these two peptides. Serum Abs against each peptide were detected with high probability from week four, through to week 15 via repeated intradermal injections of mouse nAChRα3 peptides (P1 and P2) and adjuvants. The mouse nAChRα3 shares 94% amino acid sequence identity with human nAChRα3, and, P1 and P2 peptides display high amino acid sequence homology (94 and 100%, respectively).

Clinically, autonomic dysfunction developed in the cardiovascular and digestive systems of both the P1- and P2-immunized mice. Cardiovascular autonomic dysfunction, such as reduced HR and SBP instability, developed especially from week 3 to week 5, and such dysfunction was also observed between weeks 9 and 11. Poor BW gain was confirmed at weeks 3 and 4. We observed slower intestinal transit according to the results obtained at weeks 8 and 15. The production of nAChRα3-targeting Abs is required for the development and maintenance of autonomic dysfunction in actively immunized mice. Our murine model of hAAG is characterized by subacute onset and long-term autonomic dysfunction in the chronic phase.

Moreover, a notable phenomenon observed in the present study was the development of skin lesions, particularly in P2-immunized mice. Dermal ulcers and alopecia were not reported in the rabbit model. Dermal ulcers may be adjuvant-induced dermatitis unrelated to a specific antigen. In human neuroimmunological diseases, alopecia is known to occur in patients with MG (Suzuki et al., 2005, 2013). HLA class II allele restriction or CD8 T cell infiltration are the suggested pathological mechanisms of alopecia (Suzuki et al., 2013); however, the exact pathophysiology remains unclear. In previous reports, skin lesions, including alopecia, have been suggested to occur due to dysimmune states. Furthermore, skin lesions in mice cannot be ruled out as trauma because several mice appeared restless. In the future, a more detailed evaluation of the behavior and immune response in the skin of immunized mice will be required.

Questions remain regarding whether Abs against nAChRα3 are actually pathogenic in immunized mice. In the current study, we verified the immunogenicity of P1 and P2. Pathological observation of the SCG revealed that the density of nAChRα3 subunit-positive cells in P1- and P2-immunized mice was significantly lower than that of control mice. No cell infiltration was observed in the SCG. Our results are similar to those of previous studies (Tajzoy et al., 2011). We speculated the loss of surface nAChR is due to receptor cross-linking by nAChR-specific Abs, leading to accelerated internalization and degradation (Drachman et al., 1978; Conti-Fine et al., 2006). The most probable mechanism is that nAChR Abs affects synaptic transmission in autonomic ganglia through two mechanisms: (1) Abs bind to the nAChR and functionally block it and (2) accelerated internalization and degradation of AChR molecules occur following Abs crosslinking, leading to a reduced number of nAChRs. The same mechanisms have been reported for other antibody-mediated autoimmune diseases (Hinson et al., 2012). Furthermore, mononuclear cell infiltration was not observed in the SCG of immunized mice. Based on these observations, we considered that hAAG pathogenesis was driven by antibody-mediated mechanisms, as described above. The results of GI dysmotility should be carefully interpreted, although we clearly observed slower GI transit in immunized mice. Autoimmune GI dysmotility (AGID) is an idiopathic phenomenon that has been recently shown to be associated with autoimmune dysautonomia (Dhamija et al., 2008; Meeusen et al., 2013; Flanagan et al., 2014; Mukaino et al., 2018). The occurrence of GI dysmotility has also been confirmed in EAE (Wunsch et al., 2017; Spear et al., 2018; Spear and Mawe, 2019). It has been reported that the pathomechanism of GI dysmotility in EAE caused by cellular immunity abnormality was antibody-mediated (Wunsch et al., 2017; Spear et al., 2018). In the current study, we could not assess the pathological evaluation of the intestinal myenteric ganglia. Hence, in the future, it is necessary to investigate whether this novel animal model has a unique immunological mechanism. In comparison with the passive transfer murine model, our active immunization murine model showed the same autonomic symptoms, which were considered antibody-mediated. Although the physiological analysis of our active immunization murine model was limited and further investigations are required, the results of the present study demonstrate that murine models of hAAG by both passive transfer and active immunization are now available.

The current study has several limitations that should be noted. First, we could not measure the antibody titers because only the dot blot assay was employed for the detection of Abs. Therefore, the correlation among antibody levels, severity of symptoms, and neural cell density could not be assessed. Second, we could not measure hormones, such as norepinephrine, because sufficient serum was not obtained for the mice. Although it has been highlighted that adrenergic function is impaired in rabbit models of hAAG, similar changes could not be evaluated in the hAAG murine model. Third, with regard to AAG extra-autonomic manifestations, such as brain symptoms, which we have taken up as a research topic, no inflammation-associated changes were observed in the brains of immunized mice. In future research, we should evaluate nAChRα3 expression in the central, peripheral, and enteric nervous systems. Further, we should thoroughly assess the behavior of immunized mice. Trials of adoptive transfer of lymph node T cells from active immunization mouse models or immunotherapy (intravenous immunoglobulin, plasmapheresis, etc.) need to be performed in the future (Drachman, 2003; Vernino et al., 2004; Sugihara et al., 2007).

Based on the current findings, we concluded that immunization of mice with nAChRα3 peptides P1 and P2 could induce autonomic dysfunction through the production of ganglionic nAChR-targeting Abs, similar to those observed in hAAG. The mouse nAChRα3 shares 94% amino acid sequence identity with human nAChRα3, and P1 and P2 peptides display high amino acid sequence homology (94 and 100%, respectively). In the present study, we detected Abs against P1 and P2 in the sera of patients with AAG. This assay demonstrated that approximately half of the seropositive AAG patients had Abs that reacted with the P1 and P2 proteins. These two peptides are likely to be recognized as antigens; in particular, the P1 region of human nAChRα3 was predicted to have a high binding affinity for HLA-DRB1*04:03 molecules (Supplementary Data 5). Our findings suggest that particular HLA class II molecules might be central to the induction of anti-ganglionic nAChR Abs during the autoimmune response in hAAG (Gonzalez-Gay et al., 2002; Maeda et al., 2016). Our novel murine model mimics hAAG and is a useful tool to investigate its pathophysiology. Besides serving as a tool for hAAG studies, a murine model of hAAG can be useful to elucidate the roles of nAChR in maintaining the structural and functional integrity of the nervous system. We believe that the current work lays the foundation for immunological, neurophysiological, and neuroendocrinological hAAG murine model research, which can assist in solving clinical problems (Nakane et al., 2018, 2020; Hayashi et al., 2022).

## Data availability statement

The original contributions presented in this study are included in the article/Supplementary material, further inquiries can be directed to the corresponding author.

## Ethics statement

The studies involving human participants were reviewed and approved by Ethics Committees of the Kumamoto University Hospital. The patients/participants provided their written informed consent to participate in this study. This animal study was reviewed and approved by Ethics Committee of Kumamoto University, Graduate School of Medical Sciences.

## Author contributions

SN had full access to all the data in the study and takes responsibility for the integrity of the data and accuracy of the data analysis, and supervised the study. MY and SN conceived and designed the experiments. MY, SN, EI, NT, HI, YI, YK, KT, TI, YT, AM, and SM performed the experiments. MY, SN, EI, TI, YO, and MU drafted the manuscript and statistical analysis. SN and AM obtained the funding. All authors: acquisition, analysis, or interpretation of the data.

## Footnotes

1
http://tools.iedb.org/mhcii/

## Abbreviations

AAG: autoimmune autonomic ganglionopathy
Abs: antibodies
AGID: autoimmune gastrointestinal dysmotility
ANOVA: analysis of variance
BW: body weight
CFA: Freund’s complete adjuvant
DAPI: 4’,6-diamidino-2-phenylindole
EAAN: experimental autoimmune autonomic neuropathy
EAE: experimental autoimmune encephalomyelitis
EAMG: experimental autoimmune myasthenia gravis
ELISA: enzyme-linked immunosorbent assay
GC: geometric center
GI: gastrointestinal
hAAG: human autoimmune autonomic ganglionopathy
HLA: human leukocyte antigen
HR: heart rate
HRP: horseradish peroxidase
IEDB: immune epitope database
IHC: immunohistochemistry
MG: myasthenia gravis
MHC: major histocompatibility complex
nAChR: nicotinic acetylcholine receptor
NCD: neuronal dell density
PBS: phosphate-buffered saline
SBP: systolic blood pressure
SCG: sympathetic cervical ganglia.
