# Supplementary material for: A novel murine model of autoimmune dysautonomia by α3 nicotinic acetylcholine receptor immunization
Source: Front Neurosci. 2022 Nov 23;16:1006923. doi: 10.3389/fnins.2022.1006923 (PMC9727251; doi:10.3389/fnins.2022.1006923)
Supplement: Supplementary file 1 [file Data_Sheet_1.PDF]

Supplementary data 1

Histopathological studies of the brains

After perfusion with a fixative, the brains were removed from the skull at weeks 4, 8, and 15. Two sagittal incisions were made in the brain. This allowed for systemic analysis of the olfactory bulb, cortex, striatum, thalamus, hypothalamus, hippocampus, brainstem (midbrain, pons, and medulla), and cerebellum. The resulting slides were then stained with hematoxylin and eosin. Pathological scores were assigned to the following brain areas without the knowledge of mouse strains: cerebellum, brainstem, hippocampus, striatum, and cortex. Each area of the brain was graded on a scale of 0 to 4 as follows: 0 = no pathology; 1 = no tissue destruction, but only minimal inflammation; 2 = early tissue destruction (loss of architecture) and moderate inflammation; 3 = definite tissue destruction (demyelination, parenchymal damage, cell death, neurophagia, neuronal vacuolation); and 4 = necrosis (complete loss of all tissue elements with associated cellular debris). Immunohistochemistry (IHC) using paraffin-embedded formalin-fixed tissues was performed on mouse brains as previously described. An anti-CD3 (clone SP7, Nichirei, Tokyo, Japan) antibody and an anti-Iba-1 antibody (rabbit polyclonal, WAKO, Tokyo, Japan) were used to detect T lymphocytes and microglia, respectively. An HRP-labeled anti-rabbit immunoglobulin antibody (Nichirei, Tokyo, Japan) was used as the secondary antibody. 3,3'-Diaminobenzidine was used to visualize positive signals.

REFERENCES

Nakane S, Zoecklein LJ, Gamez JD, et al. A 40-cM region on chromosome 14 plays a critical role in the development of virus persistence, demyelination, brain pathology and neurologic deficits in a murine viral model of multiple sclerosis. Brain Pathol. 2003; 13:519-33.

Hayashi K, Hasegawa Y, Takemoto Y, et al. Continuous intracerebroventricular injection of

Porphyromonas gingivalis lipopolysaccharide induces systemic organ dysfunction in a mouse model of Alzheimer's disease. Exp Gerontol. 2019; 120:1-5.
