# Supplementary material for: A novel murine model of autoimmune dysautonomia by α3 nicotinic acetylcholine receptor immunization
Source: Front Neurosci. 2022 Nov 23;16:1006923. doi: 10.3389/fnins.2022.1006923 (PMC9727251; doi:10.3389/fnins.2022.1006923)
Supplement: Supplementary file 2 [file Data_Sheet_2.PDF]

Supplementary data 2

Luciferase Immunoprecipitation System Assay for Autoantibodies against gAChR

A National Institutes of Health research group previously developed this efficient quantitative approach for analysing antibodies against human autoantigens in serum samples. We previously established that LIPS assays can be used to diagnose AAG based on the presence of immunoglobulin Gs (IgGs) to both the α3 and β4 subunits of the gAChR in serum samples. Levels of these gAChR Abs were measured at Nagasaki Kawatana Medical Center and Kumamoto University Hospital, as previously described.

To generate luciferase reporters for the human gAChR subunits, α3 and β4 (named gAChRα3-GL and gAChRβ4-GL, respectively) or full-length human gAChRα3 (P32297; Promega Corporation, Madison, WI, USA) or gAChRβ4 (P30296; Promega Corporation) was fused to a Gaussia luciferase (GL) mutant (GL^{8990}). Human embryonic kidney 293F cells (Life Technologies Corporation, Grand Island, NY, USA) were then transfected with the expression plasmid encoding either gAChRα3-GL or gAChRβ4-GL with FuGENE6 (Promega Corporation). The transfected cells were solubilized 2 days later with Tris-based saline containing 1% Triton™ X-100. To detect gAChRα3 or gAChRβ4 Abs, 100 μl of the soluble fraction containing gAChRα3-GL or gAChRβ4-GL was incubated with 15 μl of human serum for 1 h at 4°C. The fraction was then mixed with 15 μl of protein G-Sepharose (GE Healthcare, Little Chalfont, Buckinghamshire, UK) and 600 μl of phosphate-buffered saline with 3% bovine serum albumin and 0.05% Tween®-20, and the mixture was incubated for several hours at 4°C. After centrifugation and two washes with phosphate-buffered saline containing 0.05% Tween®-20, the

bioluminescence activities of the luciferase reporters in protein G-Sepharose were measured with a BioLux® GL Assay Kit (New England Biolabs, Ipswich, MA, USA) and a Lumat LB 9507 luminometer (Berthold Technologies GmbH & Co. KG, Bad Wildbad, Germany). The luminometer output was measured in relative luminescence units (RLU). Via anti-gAChRα3 and anti-gAChRβ4 Ab data from 73 healthy controls, cut-off values were calculated as means + 3 standard deviations (SD) from the mean in the previous study. Antibody levels are expressed as follows: antibody index (AI) = [measured value in the serum sample (RLU)]/[cut-off RLU value]. The healthy control value was defined as an AI of < 1.0.

To evaluate the diagnostic accuracy of this LIPS assay, we verified the cut-off points for all data collected in the previous study. Cut-off points for sensitivity and specificity, as well as ROC (receiver operating characteristic) curves, were obtained. According to the ROC curves, we confirmed the most discriminative cut-off points, and calculated their sensitivity, specificity, and positive and negative predictive values (PPV and NPV). The area under the curve (AUC) was 0.935 (95% confidence interval [CI]: 0.897–0.972) for the LIPS assay of the anti-gAChRα3 antibody. With the anti-gAChRα3 antibody cut-off point of 1.0, the sensitivity and specificity values were 50.0% (95% CI: 35.2%–64.8%) and 100.0% (95% CI: 92.5%–100.0%), respectively, while those for PPV and NPV were 100.0% and 66.7%, respectively. The AUC was 0.830 (95% CI: 0.040–0.907) for the LIPS assay of the anti-gAChRβ4 antibody. With the anti-gAChRβ4 antibody cut-off point of 1.0, the sensitivity and specificity values

were 10.0% (95% CI: 3.5%–23.8%) and 100.0% (95% CI: 92.5%–100.0%), respectively, while those for PPV and NPV were 100.0% and 52.84%, respectively.

References

Maeda Y, Migita K, Higuchi O, Mukaino A, Furukawa H, Komori A, *et al*. Association between anti-ganglionic nicotinic acetylcholine receptor (gAChR) antibodies and HLA-DRB1 alleles in the Japanese population. PLOS One. 2016; 11:e0146048.

Nakane S, Higuchi O, Koga M, Kanda T, Murata K, Suzuki T, *et al*. Clinical features of autoimmune autonomic ganglionopathy and the detection of subunit-specific autoantibodies to the ganglionic acetylcholine receptor in Japanese patients. PLOS One. 2015; 10: e0118312.

Nakane S, Mukaino A, Higuchi O, Watari M, Maeda Y, Yamakawa M, *et al*. Autoimmune autonomic ganglionopathy: an update on diagnosis and treatment. Expert Rev Neurother. 2018; 18:953-965.
