# Supplementary figures and images for: A novel murine model of autoimmune dysautonomia by α3 nicotinic acetylcholine receptor immunization
Source: Front Neurosci. 2022 Nov 23;16:1006923. doi: 10.3389/fnins.2022.1006923 (PMC9727251; doi:10.3389/fnins.2022.1006923)

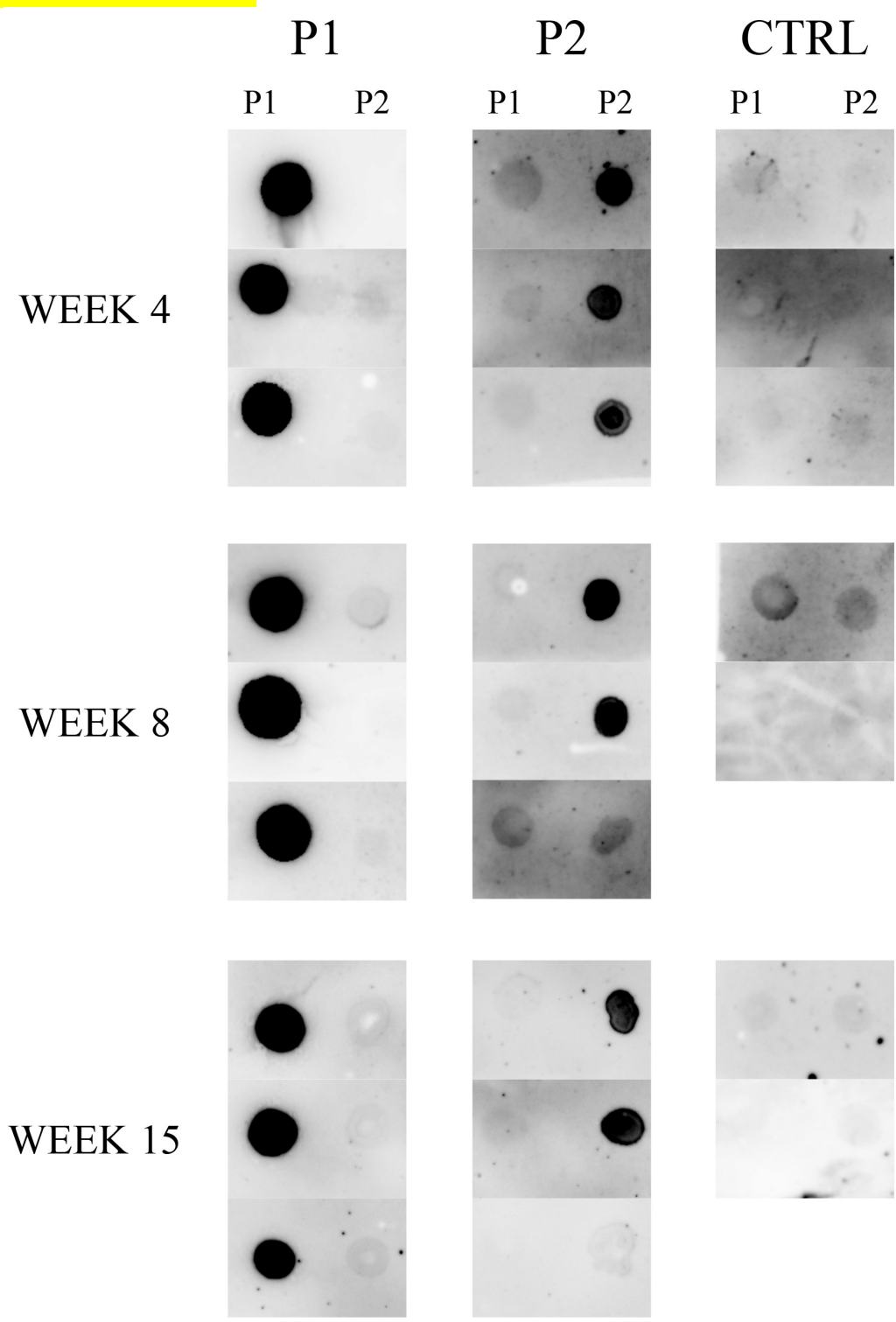

Supplement: Supplementary file 3 [file Data_Sheet_3.PDF]

High binding affinity

Percentile rank  
(IEDB recommended method)

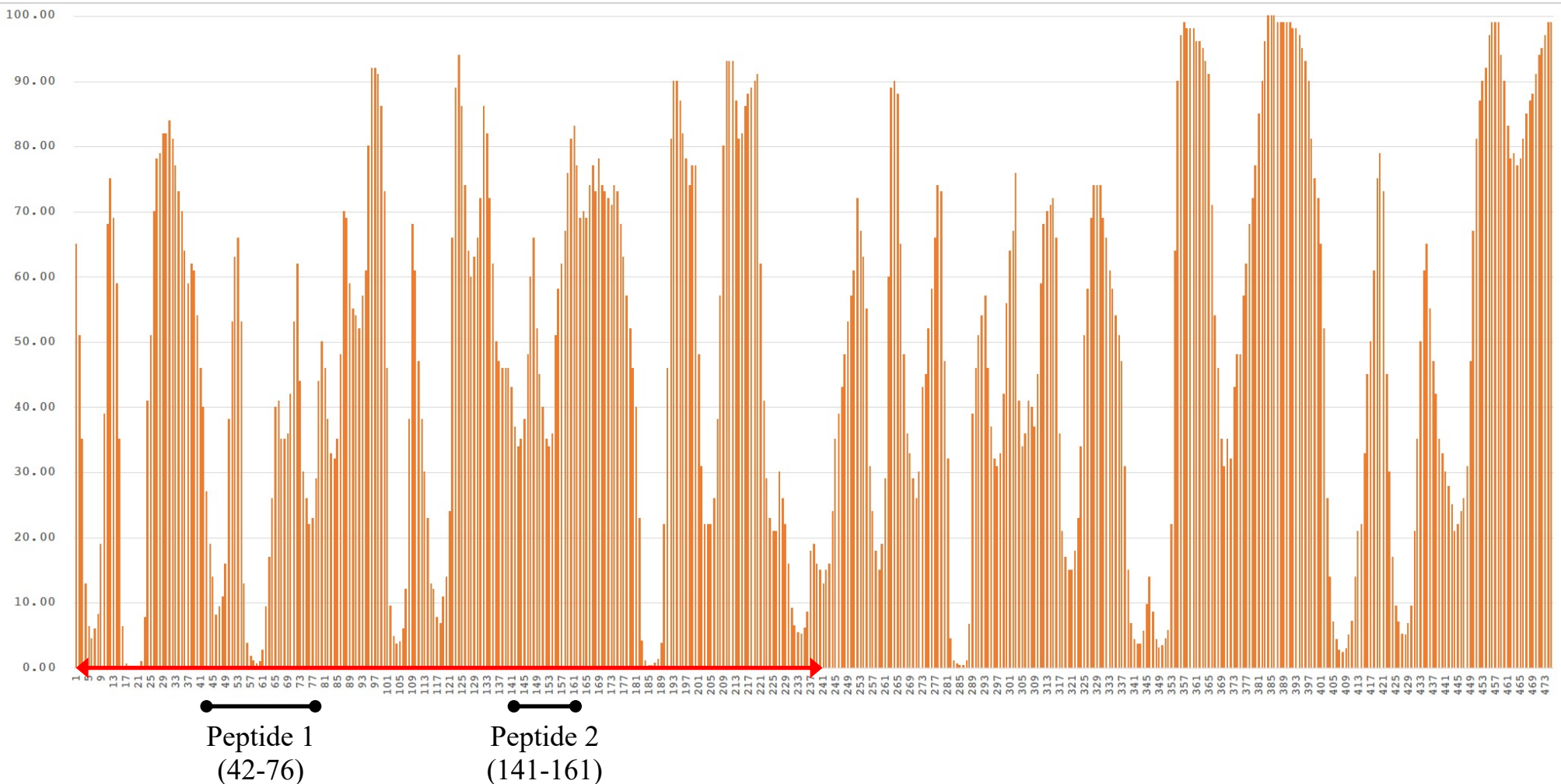

Supplement: Supplementary file 5 [file Data_Sheet_5.PDF]
