# Supplementary material for: A novel murine model of autoimmune dysautonomia by α3 nicotinic acetylcholine receptor immunization
Source: Front Neurosci. 2022 Nov 23;16:1006923. doi: 10.3389/fnins.2022.1006923 (PMC9727251; doi:10.3389/fnins.2022.1006923)
Supplement: Supplementary file 4 [file Data_Sheet_4.PDF]

Supplementary data 4.

	HR (/min))				SBP (mmHg)				%BW change			
	P1	P2	CTRL	P value	P1	P2	CTRL	P value	P1	P2	CTRL	P value
WEEK -4	492.2 ± 80.1	491.7 ± 118.4	505.9 ± 101.2	0.953	129.4 ± 31.7	155.6 ± 29.9	123.1 ± 12.9	0.052				
WEEK -3	507.8 ± 82.8	511.5 ± 88.8	569.4 ± 49.6	0.240	153.1 ± 48.3	135.1 ± 29.6	123.8 ± 18.1	0.365				
WEEK -2	506.6 ± 80.0	575.4 ± 58.7	591.3 ± 65.5	0.058	143.1 ± 20.2	126.4 ± 23.8	122.8 ± 10.6	0.100				
WEEK -1	617.1 ± 104.8	621.0 ± 57.0	596.8 ± 84.7	0.550	131.5 ± 19.6	136.9 ± 23.1	138.3 ± 24.8	0.744				
WEEK 0	607.1 ± 124.6	622.1 ± 93.2	577.6 ± 93.5	0.477	162.2 ± 31.9	150.9 ± 18.7	124.9 ± 32.8	0.080				
WEEK 1	577.3 ± 92.0	570.1 ± 168.4	652.6 ± 94.3	0.305	103.9 ± 31.3	110.1 ± 28.5	104.2 ± 12.4	0.858	4.7 ± 3.2	5.2 ± 4.3	5.6 ± 4.0	0.905
WEEK 2	403.6 ± 72.4	452.4 ± 113.7	492.4 ± 79.6	0.174	127.6 ± 46.9	120.4 ± 39.4	109.4 ± 29.8	0.669	7.0 ± 4.2	7.5 ± 3.2	10.5 ± 3.1	0.144
WEEK 3	**467.7 ± 99.2**	**446.9 ± 80.2**	631.6 ± 64.0	<0.001*	116.5 ± 19.5	**95.2 ± 11.2**	119.3 ± 18.5	0.013*	**6.2 ± 4.3**	**6.6 ± 2.6**	13.0 ± 3.5	0.001*
WEEK 4	**467.6 ± 128.3**	559.6 ± 130.9	638.1 ± 34.2	0.021*	108.1 ± 30.2	124.1 ± 31.2	124.2 ± 31.1	0.468	**7.6 ± 3.4**	**8.6 ± 2.5**	13.0 ± 2.4	0.003*
WEEK 5	471.8 ± 134.5	573.4 ± 139.6	603.6 ± 97.7	0.255	110.7 ± 18.1	**148.4 ± 22.9**	105.6 ± 13.9	0.005*	15.4 ± 4.2	16.1 ± 2.3	18.0 ± 4.7	0.570
WEEK 6	566.4 ± 125.5	627.7 ± 59.1	652.3 ± 29.1	0.295	126.8 ± 23.0	116.4 ± 10.8	114.1 ± 8.2	0.420	16.5 ± 4.8	17.9 ± 1.1	18.1 ± 4.7	0.745
WEEK 7	573.8 ± 126.4	620.9 ± 59.1	675.1 ± 23.0	0.347	124.6 ± 21.8	127.0 ± 17.7	128.4 ± 27.1	0.498	14.5 ± 3.6	15.6 ± 1.6	17.9 ± 4.1	0.268
WEEK 8	544.6 ± 97.8	644.2 ± 58.0	642.3 ± 58.5	0.112	108.5 ± 15.8	127.0 ± 6.4	118.8 ± 13.1	0.173	14.0 ± 4.6	16.8 ± 1.6	16.3 ± 4.0	0.384
WEEK 9	**509.9 ± 34.6**	626.9 ± 15.1	633.0 ± 3.9	0.003*	128.1 ± 9.5	123.5 ± 14.5	111.4 ± 8.1	0.352	16.3 ± 5.1	19.2 ± 1.9	17.3 ± 0.6	0.621
WEEK 10	**561.4 ± 40.2**	673.8 ± 45.4	655.6 ± 13.9	0.036*	110.8 ± 12.6	108.1 ± 5.6	116.8 ± 2.8	0.592	15.7 ± 4.3	19.4 ± 2.1	17.0 ± 2.8	0.511
WEEK 11	527.0 ± 88.6	658.6 ± 25.5	598.7 ± 60.0	0.130	123.8 ± 4.6	**105.1 ± 6.3**	113.2 ± 0.4	0.016*	12.1 ± 3.0	18.2 ± 1.4	14.8 ± 5.5	0.161
WEEK 12	515.2 ± 73.6	615.9 ± 51.4	607.0 ± 18.6	0.179	140.8 ± 27.9	117.9 ± 20.0	122.2 ± 5.5	0.461	18.2 ± 4.5	23.4 ± 1.6	21.4 ± 4.1	0.271
WEEK 13	640.0 ± 48.0	705.0 ± 13.4	679.9 ± 27.5	0.152	158.7 ± 32.0	117.5 ± 2.7	137.5 ± 21.1	0.174	16.2 ± 3.2	21.5 ± 1.0	19.5 ± 7.0	0.286
WEEK 14	522.2 ± 154.5	678.2 ± 40.9	712.1 ± 10.3	0.160	127.4 ± 21.8	108.5 ± 8.5	131.3 ± 3.8	0.256	10.2 ± 6.3	17.2 ± 3.9	18.8 ± 5.3	0.221
WEEK 15	498.7 ± 125.9	687.8 ± 20.7	689.9 ± 15.6	0.061	110.6 ± 10.4	127.0 ± 8.4	110.4 ± 9.1	0.147	12.4 ± 8.8	17.3 ± 3.2	9.8 ± 1.5	0.415

HR, heart rate; SBP, systolic blood pressure; BW, body weight; P1, P1 immunized mice; P2, P2 immunized mice; CTRL, control mice

Red frame means immunization with each 200 μg nAChRα3 peptide (P1 and P2) at WEEK 0 and 1.

Black dotted frame means mice from each group were sacrificed at WEEK 4, 8, and 15.

Blue letters indicate the value is significantly lower than the values of other groups.

Red letters indicate the value is significantly higher than the values of other groups.
